# *catena*-Poly[[(2,2′-dimethyl-4,4′-bi-1,3-thia­zole-κ^2^ *N*,*N*′)cadmium]-di-μ-bromido]

**DOI:** 10.1107/S1600536811020861

**Published:** 2011-06-11

**Authors:** Anita Abedi, Forugh Rezaei

**Affiliations:** a Department of Chemistry, North Tehran Branch, Islamic Azad University, Tehran, Iran

## Abstract

In the title coordination polymer, [CdBr_2_(C_8_H_8_N_2_S_2_)]_*n*_, the Cd^II^ atom is six-coordinated in a distorted octa­hedral geometry by two N atoms from a 2,2′-dimethyl-4,4′-bi-1,3-thia­zole ligand and four bridging Br atoms. The bridging function of the Br atoms leads to a chain structure along [100]. Inter­chain C—H⋯Br hydrogen bonds and π–π contacts between the thia­zole rings [centroid–centroid distances = 3.810 (5) and 3.679 (5) Å] are observed.

## Related literature

For metal complexes with 2,2′-dimethyl-4,4′-bi-1,3-thia­zole, see: Abedi (2011 ▶); Abedi & Yahyazade Bali (2010 ▶); Al-Hashemi *et al.* (2009 ▶, 2010 ▶); Khavasi *et al.* (2008 ▶); Notash *et al.* (2008 ▶, 2009 ▶); Safari *et al.* (2009 ▶).
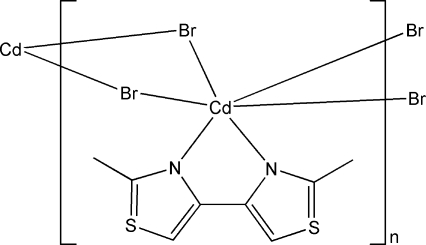


## Experimental

### 

#### Crystal data

[CdBr_2_(C_8_H_8_N_2_S_2_)]
*M* *_r_* = 468.51
Triclinic, 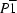

*a* = 7.1936 (10) Å
*b* = 9.5775 (11) Å
*c* = 10.4218 (14) Å
α = 112.714 (9)°
β = 104.149 (11)°
γ = 92.68 (1)°
*V* = 634.20 (16) Å^3^
*Z* = 2
Mo *K*α radiation
μ = 8.32 mm^−1^
*T* = 298 K
0.28 × 0.18 × 0.13 mm

#### Data collection

Bruker APEX CCD diffractometer
Absorption correction: multi-scan (*SADABS*; Sheldrick, 1996 ▶) *T*_min_ = 0.180, *T*_max_ = 0.340
7003 measured reflections
3400 independent reflections
2495 reflections with *I* > 2σ(*I*)
*R*_int_ = 0.160

#### Refinement

*R*[*F* ^2^ > 2σ(*F* ^2^)] = 0.081
*wR*(*F* ^2^) = 0.232
*S* = 1.05
3400 reflections
136 parameters
H-atom parameters constrained
Δρ_max_ = 2.22 e Å^−3^
Δρ_min_ = −4.78 e Å^−3^

### 

Data collection: *SMART* (Bruker, 2007 ▶); cell refinement: *SAINT* (Bruker, 2007 ▶); data reduction: *SAINT*; program(s) used to solve structure: *SHELXTL* (Sheldrick, 2008 ▶); program(s) used to refine structure: *SHELXTL*; molecular graphics: *ORTEP-3* (Farrugia, 1997 ▶); software used to prepare material for publication: *WinGX* (Farrugia, 1999 ▶).

## Supplementary Material

Crystal structure: contains datablock(s) I, global. DOI: 10.1107/S1600536811020861/hy2437sup1.cif

Structure factors: contains datablock(s) I. DOI: 10.1107/S1600536811020861/hy2437Isup2.hkl

Additional supplementary materials:  crystallographic information; 3D view; checkCIF report

## Figures and Tables

**Table 1:** Selected bond lengths (Å)

Cd1—N1	2.435 (7)
Cd1—N2	2.372 (7)
Cd1—Br1	2.7112 (11)
Cd1—Br2^i^	2.7640 (12)
Cd1—Br1^ii^	2.8362 (12)
Cd1—Br2	2.7845 (12)

Symmetry codes: (i) 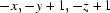; (ii) 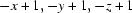.

**Table 2:** Hydrogen-bond geometry (Å, °)

*D*—H⋯*A*	*D*—H	H⋯*A*	*D*⋯*A*	*D*—H⋯*A*
C3—H3⋯Br1^iii^	0.93	2.87	3.754 (11)	159
C6—H6⋯Br1^iii^	0.93	2.86	3.772 (10)	166
C8—H8*C*⋯Br2	0.96	2.74	3.681 (16)	167

Symmetry code: (iii) 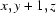.

**Fig. 1.**
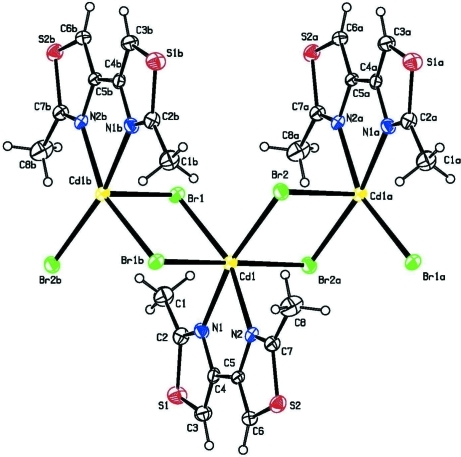
Part of the chain structure of the title compound. Displacement ellipsoids are drawn at the 50% probability level. [Symmetry codes: (a) -x, 1-y, 1-z; (b) 1-x, 1-y, 1-z.]

**Fig. 2.**
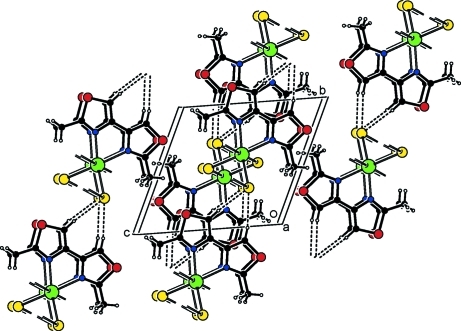
The packing diagram for the title compound.

## supplementary crystallographic information

### Comment

Recently, we reported the synthesis and crystal structures of [HgI_2_(dm4bt)] (Abedi & Yahyazade Bali, 2010) and [HgBr_2_(dm4bt)] (Abedi, 2011) (dm4bt = 2,2'-dimethyl-4,4'-bi-1,3-thiazole). Dm4bt is a good bidentate ligand, and numerous complexes with dm4bt have been prepared, such as that of zinc (Khavasi *et al.*, 2008), thallium (Notash *et al.*, 2008), cadmium (Notash *et al.*, 2009), mercury (Safari *et al.*, 2009) and copper (Al-Hashemi *et al.*, 2009, 2010). For further investigation of dm4bt, we synthesized the title complex and report herein its crystal structure.

In the title coordination polymer (Fig. 1), the Cd^II^ atom is six-coordinated in a distorted octahedral geometry by two N atoms from a dm4bt ligand and four bridging Br atoms (Table 1). The bridging function of the bromide atoms leads to a one-dimensional chain structure along [1 0 0] (Fig. 2).

In the crystal structure, intermolecular C—H···Br hydrogen bonds (Table 2) and π–π contacts (Fig. 2) between the thiazole rings, *Cg*4···*Cg*5^i^ = 3.810 (5) and *Cg*5···*Cg*5^ii^ = 3.679 (5) Å [symmetry codes: (i) 1-x, 2-y, 1-z; (ii) -x, 2-y, 1-z. *Cg*4 and *Cg*5 are the centroids of the S1/C2/N1/C4/C3 and S2/C6/C5/N2/C7 rings], stabilize the structure.

### Experimental

For the preparation of the title compound, a solution of dm4bt (0.26 g, 1.3 mmol) in methanol (15 ml) was added to a solution of CdBr_2_.4H_2_O (0.44 g, 1.3 mmol) in methanol (15 ml) at room temperature. The suitable crystals for X-ray diffraction experiment were obtained by methanol diffusion into a colorless solution in DMF. The crystals were isolated after one week (yield: 0.45 g, 73.9%).

### Refinement

H atoms were positioned geometrically and refined as riding atoms, with C—H = 0.93 (CH) and 0.96 (CH_3_) Å and with *U*_iso_(H) = 1.2(1.5 for methyl)*U*_eq_(C). The highest residual electron density was found at 0.89 Å from Cd1 atom and the deepest hole at 0.88 Å from Cd1 atom.

### Crystal data

**Table:**

[CdBr_2_(C_8_H_8_N_2_S_2_)]	*Z* = 2
*M**_r_* = 468.51	*F*(000) = 440
Triclinic, *P*1	*D*_x_ = 2.454 Mg m^−^^3^
Hall symbol: -P 1	Mo *K*α radiation, λ = 0.71073 Å
*a* = 7.1936 (10) Å	Cell parameters from 7003 reflections
*b* = 9.5775 (11) Å	θ = 2.2–29.2°
*c* = 10.4218 (14) Å	µ = 8.32 mm^−^^1^
α = 112.714 (9)°	*T* = 298 K
β = 104.149 (11)°	Block, colorless
γ = 92.68 (1)°	0.28 × 0.18 × 0.13 mm
*V* = 634.20 (16) Å^3^	

### Data collection

**Table:**

Bruker APEX CCD diffractometer	3400 independent reflections
Radiation source: fine-focus sealed tube	2495 reflections with *I* > 2σ(*I*)
graphite	*R*_int_ = 0.160
φ and ω scans	θ_max_ = 29.2°, θ_min_ = 2.2°
Absorption correction: multi-scan (*SADABS*; Sheldrick, 1996)	*h* = −9→9
*T*_min_ = 0.180, *T*_max_ = 0.340	*k* = −13→12
7003 measured reflections	*l* = −14→14

### Refinement

**Table:**

Refinement on *F*^2^	Primary atom site location: structure-invariant direct methods
Least-squares matrix: full	Secondary atom site location: difference Fourier map
*R*[*F*^2^ > 2σ(*F*^2^)] = 0.081	Hydrogen site location: inferred from neighbouring sites
*wR*(*F*^2^) = 0.232	H-atom parameters constrained
*S* = 1.05	*w* = 1/[σ^2^(*F*_o_^2^) + (0.149*P*)^2^] where *P* = (*F*_o_^2^ + 2*F*_c_^2^)/3
3400 reflections	(Δ/σ)_max_ = 0.005
136 parameters	Δρ_max_ = 2.22 e Å^−^^3^
0 restraints	Δρ_min_ = −4.78 e Å^−^^3^

### Fractional atomic coordinates and isotropic or equivalent isotropic displacement parameters (Å^2^)

**Table:**

	*x*	*y*	*z*	*U*_iso_*/*U*_eq_	
C1	0.2848 (18)	0.4579 (13)	0.0750 (12)	0.050 (3)	
H1A	0.1742	0.3946	0.0710	0.060*	
H1B	0.4015	0.4317	0.1221	0.060*	
H1C	0.2854	0.4415	−0.0218	0.060*	
C2	0.2745 (13)	0.6214 (11)	0.1579 (9)	0.0338 (18)	
C3	0.2515 (14)	0.8908 (11)	0.2347 (10)	0.0362 (19)	
H3	0.2437	0.9918	0.2478	0.043*	
C4	0.2597 (11)	0.8389 (10)	0.3383 (9)	0.0277 (16)	
C5	0.2502 (11)	0.9274 (9)	0.4849 (9)	0.0262 (15)	
C6	0.2499 (13)	1.0824 (10)	0.5464 (10)	0.0342 (18)	
H6	0.2571	1.1478	0.5004	0.041*	
C7	0.2327 (13)	0.9521 (11)	0.7021 (10)	0.0332 (18)	
C8	0.220 (2)	0.9124 (16)	0.8265 (12)	0.057 (3)	
H8C	0.2207	0.8045	0.7987	0.068*	
H8B	0.1028	0.9388	0.8511	0.068*	
H8A	0.3300	0.9686	0.9089	0.068*	
N1	0.2720 (11)	0.6838 (9)	0.2955 (8)	0.0298 (14)	
N2	0.2379 (10)	0.8540 (8)	0.5760 (8)	0.0282 (14)	
Cd1	0.24677 (9)	0.58678 (7)	0.47579 (7)	0.0302 (2)	
Br1	0.36362 (13)	0.31498 (11)	0.34631 (11)	0.0364 (3)	
Br2	0.14395 (13)	0.49070 (11)	0.67291 (10)	0.0353 (3)	
S1	0.2574 (4)	0.7476 (3)	0.0763 (2)	0.0420 (6)	
S2	0.2347 (4)	1.1375 (3)	0.7182 (2)	0.0364 (5)	

### Atomic displacement parameters (Å^2^)

**Table:**

	*U*^11^	*U*^22^	*U*^33^	*U*^12^	*U*^13^	*U*^23^
C1	0.056 (6)	0.038 (5)	0.047 (6)	0.009 (5)	0.019 (5)	0.007 (4)
C2	0.034 (4)	0.035 (5)	0.030 (4)	0.007 (4)	0.009 (3)	0.011 (3)
C3	0.041 (5)	0.032 (5)	0.038 (4)	0.009 (4)	0.013 (4)	0.016 (4)
C4	0.023 (4)	0.033 (4)	0.031 (4)	0.010 (3)	0.012 (3)	0.014 (3)
C5	0.022 (4)	0.021 (4)	0.036 (4)	0.005 (3)	0.008 (3)	0.012 (3)
C6	0.035 (5)	0.026 (4)	0.045 (5)	0.007 (3)	0.016 (4)	0.016 (4)
C7	0.032 (4)	0.030 (4)	0.039 (4)	0.007 (3)	0.011 (3)	0.014 (4)
C8	0.084 (9)	0.059 (7)	0.037 (5)	0.032 (7)	0.026 (5)	0.021 (5)
N1	0.031 (4)	0.031 (4)	0.031 (3)	0.004 (3)	0.013 (3)	0.013 (3)
N2	0.025 (3)	0.028 (3)	0.035 (3)	0.011 (3)	0.011 (3)	0.014 (3)
Cd1	0.0309 (4)	0.0278 (4)	0.0387 (4)	0.0106 (2)	0.0152 (3)	0.0168 (3)
Br1	0.0350 (5)	0.0295 (5)	0.0480 (5)	0.0114 (4)	0.0154 (4)	0.0163 (4)
Br2	0.0306 (5)	0.0426 (6)	0.0383 (5)	0.0062 (4)	0.0109 (4)	0.0220 (4)
S1	0.0533 (15)	0.0470 (14)	0.0313 (11)	0.0098 (11)	0.0142 (10)	0.0205 (10)
S2	0.0375 (12)	0.0300 (11)	0.0378 (11)	0.0091 (9)	0.0129 (9)	0.0082 (9)

### Geometric parameters (Å, °)

**Table:**

C1—C2	1.484 (14)	C6—H6	0.9300
C1—H1A	0.9600	C7—N2	1.298 (11)
C1—H1B	0.9600	C7—C8	1.505 (14)
C1—H1C	0.9600	C7—S2	1.717 (10)
C2—N1	1.329 (11)	C8—H8C	0.9600
C2—S1	1.719 (10)	C8—H8B	0.9600
C3—C4	1.343 (13)	C8—H8A	0.9600
C3—S1	1.703 (9)	Cd1—N1	2.435 (7)
C3—H3	0.9300	Cd1—N2	2.372 (7)
C4—N1	1.390 (11)	Cd1—Br1	2.7112 (11)
C4—C5	1.454 (11)	Cd1—Br2^i^	2.7640 (12)
C5—C6	1.372 (12)	Cd1—Br1^ii^	2.8362 (12)
C5—N2	1.397 (11)	Cd1—Br2	2.7845 (12)
C6—S2	1.693 (10)		
			
C2—C1—H1A	109.5	H8C—C8—H8A	109.5
C2—C1—H1B	109.5	H8B—C8—H8A	109.5
H1A—C1—H1B	109.5	C2—N1—C4	110.3 (8)
C2—C1—H1C	109.5	C2—N1—Cd1	135.3 (7)
H1A—C1—H1C	109.5	C4—N1—Cd1	114.0 (5)
H1B—C1—H1C	109.5	C7—N2—C5	110.7 (8)
N1—C2—C1	125.4 (9)	C7—N2—Cd1	134.0 (6)
N1—C2—S1	113.9 (7)	C5—N2—Cd1	115.2 (5)
C1—C2—S1	120.7 (7)	N2—Cd1—N1	71.9 (2)
C4—C3—S1	111.2 (7)	N2—Cd1—Br1	161.12 (17)
C4—C3—H3	124.4	N1—Cd1—Br1	96.12 (18)
S1—C3—H3	124.4	N2—Cd1—Br2^i^	94.19 (17)
C3—C4—N1	114.9 (7)	N1—Cd1—Br2^i^	84.58 (18)
C3—C4—C5	126.3 (8)	Br1—Cd1—Br2^i^	99.26 (4)
N1—C4—C5	118.8 (7)	N2—Cd1—Br2	103.26 (18)
C6—C5—N2	114.2 (8)	N1—Cd1—Br2	168.62 (18)
C6—C5—C4	125.9 (8)	Br1—Cd1—Br2	91.01 (4)
N2—C5—C4	120.0 (7)	Br2^i^—Cd1—Br2	85.52 (3)
C5—C6—S2	110.2 (7)	N2—Cd1—Br1^ii^	82.02 (17)
C5—C6—H6	124.9	N1—Cd1—Br1^ii^	98.39 (18)
S2—C6—H6	124.9	Br1—Cd1—Br1^ii^	85.48 (4)
N2—C7—C8	124.8 (9)	Br2^i^—Cd1—Br1^ii^	174.15 (3)
N2—C7—S2	114.6 (7)	Br2—Cd1—Br1^ii^	90.99 (3)
C8—C7—S2	120.6 (7)	Cd1—Br1—Cd1^ii^	94.52 (4)
C7—C8—H8C	109.5	Cd1^i^—Br2—Cd1	94.48 (3)
C7—C8—H8B	109.5	C3—S1—C2	89.8 (4)
H8C—C8—H8B	109.5	C6—S2—C7	90.3 (4)
C7—C8—H8A	109.5		
			
S1—C3—C4—N1	0.4 (10)	C7—N2—Cd1—Br2	14.8 (8)
S1—C3—C4—C5	−177.4 (7)	C5—N2—Cd1—Br2	−169.0 (5)
C3—C4—C5—C6	−6.7 (14)	C7—N2—Cd1—Br1^ii^	−74.4 (8)
N1—C4—C5—C6	175.7 (8)	C5—N2—Cd1—Br1^ii^	101.8 (5)
C3—C4—C5—N2	172.6 (8)	C2—N1—Cd1—N2	−174.0 (9)
N1—C4—C5—N2	−5.1 (11)	C4—N1—Cd1—N2	−2.8 (5)
N2—C5—C6—S2	0.1 (9)	C2—N1—Cd1—Br1	21.0 (9)
C4—C5—C6—S2	179.4 (7)	C4—N1—Cd1—Br1	−167.8 (5)
C1—C2—N1—C4	−179.4 (9)	C2—N1—Cd1—Br2^i^	−77.8 (8)
S1—C2—N1—C4	−1.2 (10)	C4—N1—Cd1—Br2^i^	93.4 (6)
C1—C2—N1—Cd1	−8.0 (15)	C2—N1—Cd1—Br2	−107.5 (11)
S1—C2—N1—Cd1	170.3 (5)	C4—N1—Cd1—Br2	63.8 (12)
C3—C4—N1—C2	0.5 (11)	C2—N1—Cd1—Br1^ii^	107.3 (8)
C5—C4—N1—C2	178.5 (7)	C4—N1—Cd1—Br1^ii^	−81.5 (6)
C3—C4—N1—Cd1	−172.9 (6)	N2—Cd1—Br1—Cd1^ii^	48.6 (6)
C5—C4—N1—Cd1	5.0 (9)	N1—Cd1—Br1—Cd1^ii^	97.98 (18)
C8—C7—N2—C5	−179.7 (10)	Br2^i^—Cd1—Br1—Cd1^ii^	−176.54 (3)
S2—C7—N2—C5	1.9 (10)	Br2—Cd1—Br1—Cd1^ii^	−90.92 (4)
C8—C7—N2—Cd1	−3.4 (15)	Br1^ii^—Cd1—Br1—Cd1^ii^	0.0
S2—C7—N2—Cd1	178.2 (4)	N2—Cd1—Br2—Cd1^i^	93.26 (18)
C6—C5—N2—C7	−1.3 (10)	N1—Cd1—Br2—Cd1^i^	29.7 (9)
C4—C5—N2—C7	179.4 (7)	Br1—Cd1—Br2—Cd1^i^	−99.21 (4)
C6—C5—N2—Cd1	−178.4 (6)	Br2^i^—Cd1—Br2—Cd1^i^	0.0
C4—C5—N2—Cd1	2.3 (9)	Br1^ii^—Cd1—Br2—Cd1^i^	175.30 (3)
C7—N2—Cd1—N1	−176.0 (9)	C4—C3—S1—C2	−0.8 (7)
C5—N2—Cd1—N1	0.2 (5)	N1—C2—S1—C3	1.2 (7)
C7—N2—Cd1—Br1	−123.4 (8)	C1—C2—S1—C3	179.5 (9)
C5—N2—Cd1—Br1	52.8 (9)	C5—C6—S2—C7	0.8 (7)
C7—N2—Cd1—Br2^i^	101.1 (8)	N2—C7—S2—C6	−1.6 (8)
C5—N2—Cd1—Br2^i^	−82.7 (5)	C8—C7—S2—C6	179.9 (9)

Symmetry codes: (i) −*x*, −*y*+1, −*z*+1; (ii) −*x*+1, −*y*+1, −*z*+1.

### Hydrogen-bond geometry (Å, °)

**Table:**

*D*—H···*A*	*D*—H	H···*A*	*D*···*A*	*D*—H···*A*
C3—H3···Br1^iii^	0.93	2.87	3.754 (11)	159
C6—H6···Br1^iii^	0.93	2.86	3.772 (10)	166
C8—H8C···Br2	0.96	2.74	3.681 (16)	167

Symmetry codes: (iii) *x*, *y*+1, *z*.
